# Adiponectin as Well as Compressive Forces Regulate *in vitro* β-Catenin Expression on Cementoblasts via Mitogen-Activated Protein Kinase Signaling Activation

**DOI:** 10.3389/fcell.2021.645005

**Published:** 2021-04-28

**Authors:** Jiawen Yong, Julia von Bremen, Gisela Ruiz-Heiland, Sabine Ruf

**Affiliations:** Department of Orthodontics, Faculty of Medicine, Justus Liebig University Giessen, Giessen, Germany

**Keywords:** adiponectin, MAPK pathway, β-catenin, cementoblast, compression

## Abstract

We aimed to investigate the molecular effect that adiponectin exerts on cementoblasts especially in the presence of compressive forces. OCCM-30 cells (M. Somerman, NIH, NIDCR, United States) were used. Real-time reverse transcriptase–polymerase chain reaction (RT-PCR) and western blots were employed to verify if the mRNA and protein levels of adiponectin receptors (AdipoRs), mitogen-activated protein kinase (MAPK), and β-catenin signaling were influenced by compressive forces or adiponectin. Moreover, siRNAs targeting *P38*α, *JNK1*, *ERK1*, *ERK2*, and *AdipoRs* as well as pharmacological MAPK inhibition were performed. We found that compressive forces increase the expression of *AdipoRs*. Adiponectin and compression up-regulate *P38*α,*JNK1*, *ERK1*, and *ERK2* as well as β-*catenin* gene expression. Western blots showed that co-stimuli activate the MAPK and β-catenin signaling pathways. MAPK inhibition alters the compression-induced β-catenin activation and the siRNAs targeting *AdipoRs*, *P38*α, and *JNK1*, showing the interaction of single MAPK molecules and β-catenin signaling in response to compression or adiponectin. Silencing by a dominantly negative version of *P38*α and *JNK1* attenuates adiponectin-induced TCF/LEF reporter activation. Together, we found that light compressive forces activate β-catenin and MAPK signaling pathways. Adiponectin regulates β-catenin signaling principally by inactivating the GSK-3β kinase activity. β-Catenin expression was partially inhibited by MAPK blockade, indicating that MAPK plays a crucial role regulating β-catenin during cementogenesis. Moreover, adiponectin modulates GSK-3β and β-catenin mostly through AdipoR1. P38α is a key connector between β-catenin, TCF/LEF transcription, and MAPK signaling pathway.

## Introduction

Orthodontically induced inflammatory root resorption (OIIRR) is one of the possible iatrogenic side effects of orthodontic therapy, which has been the subject of many research projects around the world (Yassir et al., 2020). OIIRR is defined as the pathologic removal of cementum and dentin (Kanas and Kanas, 2011). Cementoblasts, the cells comprising the cellular component of cementum, have a similar gene expression pattern as osteoblasts, including *glycogen synthase kinase-3*β (*GSK-3*β), β-*catenin*, *osteocalcin* (*OCN*), and *osteoprotegerin* (*OPG*) (Saygin et al., 2000; Matthews et al., 2016).

It has been highlighted that β-catenin signaling regulates cementum homeostasis, and a down-regulation of WNT causes root resorptions (Lim et al., 2014). Mechanical compression of the periodontium is one of the numerous conditions occurring during orthodontic tooth movement (Memmert et al., 2019). As part of the periodontium, the cementum is often compressed through orthodontic force application. Thus, cementoblasts become mechanically deformed and start repairing the acellular cementum by replenishing it with cellular mineralized cementum (Diercke et al., 2014). Some research into the biologic regulation of the cementum repairing process have revealed that the regulation of β-catenin signaling is critical for normal cementum remodeling under constant mechanical loading (Lim et al., 2014; Sindhavajiva et al., 2018).

During orthodontic force application, compressive forces also induce human mandibular-derived osteoblast differentiation via WNT/β-catenin signaling (Sindhavajiva et al., 2018). WNT signaling was reported to inhibit cementoblast differentiation and promote their proliferation (Nemoto et al., 2009). Multiple signaling pathways participate in this process (Bikkavilli and Malbon, 2009). Recent advances suggest a possible intersection cross-reacting network: the β-catenin signaling in turn is mediated by the mitogen-activated protein kinase (MAPK) signaling pathway (Bikkavilli et al., 2008; Thornton et al., 2008). The P38 MAPK pathway is known to regulate osteoblast (Thouverey and Caverzasio, 2016) and cementoblast differentiation (Sanchavanakit et al., 2015). Bikkavilli et al. (2008) demonstrated that P38 MAPK inactivates GSK-3β by direct phosphorylation at its C terminus, leading to the activation of β-catenin signaling (Bikkavilli et al., 2008).

Adiponectin, a secreted protein produced mainly by adipocytes (Scherer et al., 1995), binds to its two receptors (AdipoR1 and AdipoR2) (Yamauchi et al., 2003). Adiponectin and its two receptors were reported to be expressed in primary human osteoblasts (Lin et al., 2014) and cementoblasts (Yong et al., 2020), which act as an important signaling link between fat and body weight to bone density (Berner et al., 2004). Unlike other adipokines such as leptin, the concentration of circulating adiponectin in obese human individuals is significantly lower than in non-obese (Naot et al., 2017). Recently, we elucidated the expression of adiponectin receptors on OCCM-30 cells and revealed that adiponectin has a pro-cementogenesis effect on cementoblasts partly through the MAPK signaling pathway (Yong et al., 2020). However, some research reveal that adiponectin has emerged as a key mediator regulating the anti-inflammatory and anti-apoptotic effects of biological activities (Tilg and Wolf, 2005; Sun and Chen, 2010).

It is now of interest how the interaction between the MAPK pathway and β-catenin signaling occurs. Additionally, the mechanism of adiponectin regulation in cementoblasts remains unclear. The aim of the current study is to assess whether β-catenin signaling modulation is regulated via the activation of MAPK signaling. Furthermore, it is aimed to identify the molecular mechanism by which adiponectin-induced MAPK interacts with compression-induced β-catenin.

## Materials and Methods

### Cell Culture

The OCCM-30 cementoblast cell line was kindly provided by Prof. M. Somerman (NIH, NIDCR, Bethesda, Maryland) and maintained in α-MEM (11095-080, Gibco) containing 10% fetal bovine serum (FBS) (10270-106, Gibco) and 1% penicillin/streptomycin (15140-122, Gibco) and incubated in a humidified atmosphere of 5% CO_2_ at 37°C. Cells were stimulated using different concentrations of mouse adiponectin/Acrp30/ADIPOQ protein (His Tag) from Sino Biological Inc. (Cat. No.: 113 50636-M08H) with or without compressive force application as described by Kanzaki et al. (2002) and Proff et al. (2014). To achieve this purpose, 33-mm diameter glass cylinders with pulled surfaces were made by Reichmann Feinoptik Inc. (Brokdorf, Germany). Cylinders of different volumes were fabricated in order to reach pressure forces of 1.2, 2.4, and 3.6 gf/cm^2^, respectively. The cells were seeded into six-well plates at a density of 3 × 10^4^ cells/well until confluence and covered with the glass cylinders afterward.

To induce cementogenesis, the cell culture medium was supplemented with 10 mM β-glycerophosphate (#35675, Calbiochem) and 50 μg/ml ascorbic acid (6288.1, Roth).

Inhibitors for P38 (SB203580) (#tlrl-sb20, InvivoGen), ERK1/2 (FR180204) (#328007, Calbiochem), and JNK (SP600125) (#tlrl-sp60, InvivoGen) were used.

### RNA Silencing

The siRNAs targeting mouse AdipoR1 (SI00890295), AdipoR2 (SI00890323), MAPK1 (SI02672117), MAPK3 (SI01300579), MAPK8 (SI1300691), MAPK14 (SI01300523), negative control (1027280), and cell death control (SI04939025) were purchased from QIAGEN Inc. siRNAs were incubated with 12 μl HiPerFect^@^ Transfection Reagent (301705, QIAGEN) in 100 μl Opti-MEM medium (31985-062, Gibco) at room temperature for 10 min, and then, each transfection mixture was added into 2.3 ml growth medium in the six-well plate in which OCCM-30 cells were cultured at 60–70% confluence. After siRNA transfection for 24 h, the cells were kept in starvation medium [(α-MEM (11095-080, Gibco) containing 0.5% FBS (10270-106, Gibco) and 1% penicillin/streptomycin (15140-122, Gibco)] for 2 h, and afterward, 100 ng/ml adiponectin (Cat. No.: 50636-M08H, Sino Biological Inc.) was added.

### Real-Time Reverse Transcriptase–Polymerase Chain Reaction

Cells were grown to confluency and kept overnight in starvation medium [α-MEM (11095-080, Gibco) containing 0.5% FBS (10270-106, Gibco) and 1% penicillin/streptomycin (15140-122, Gibco)]. Afterward, cells were either stimulated with adiponectin (100 ng/ml) (Cat. No.: 50636-M08H, Sino Biological Inc.) or cultivated under compression (1.2, 2.4, and 3.6 gf/cm^2^). Total RNA was extracted using NucleoSpin^®^ RNA Kit (740955.50, MACHEREY-NAGEL). RNA concentrations were measured using a spectrophotometer (NanoDrop 2000, Thermo Scientific). Using commercial innuSCRIPT Reverse Transcriptase kit (845-RT-6000100, Analytik Jena), 1.0 μg RNA was transcribed, and 1.0 μl of the resulting cDNA was used at the final reaction volume of 20 μl/well in a CFX96^TM^ Real-Time System Cycler (Bio-Rad). Real-time reverse transcriptase–polymerase chain reaction (RT-PCR) amplification was carried out using the SsoAdvanced^TM^ Universal SYBR^@^ Green Supermix (1723271, Bio-Rad). The primers for mouse AdipoR1&2 (qMmuCID0023619 and qMmuCID0010157) and MAPK3 (qMmuCED0025043) were purchased from Bio-Rad. The primers for MAPK1, MAPK8, MAPK14, GSK-3β, and β-catenin were purchased from Eurofins Genomics Inc. Primer sets used in the analysis are listed in Supplementary Table 1. GAPDH (qMmuCED0027497, Bio-Rad) and β-actin (qMmuCED0027505, Bio-Rad) served as housekeeping genes. Results were analyzed using the Bio-Rad CFX Manager 3.1 software.

### Protein Extraction and Western Blot

The OCCM-30 cells were lysed in RIPA buffer (89901, Thermo Scientific) supplied with 3% protease inhibitor (78442, Thermo Scientific). The insoluble material was removed by centrifugation at 14,000 rpm/min for 15 min. Protein concentrations were measured using Pierce^TM^ BCA Protein Assay Kit (23225, Thermo Scientific) on a DR/2000 Spectrophotometer (#4480000, HACH). Then, 20 μg lysate/lane was diluted in a sample loading buffer (#G031, abm) and separated by 10–12% SDS-PAGE gels and then the resolved proteins were transferred electrophoretically to nitrocellulose membranes (1704271, Bio-Rad). Protein loading was verified by Ponceau S staining (6226-79-5, Sigma). Membranes were blocked with 5% non-fat milk (T145.1, ROTH) for 1 h at room temperature and further incubated with the primary antibodies for ERK1/2 (1:1,000, MBS8241746, BIOZOL); phospho-ERK1/2 (p44/42, Thr202/Tyr204) (1:1,000, #4370, Cell Signaling Technology); p54/p56 JNK (1:1,000, #9252, Cell Signaling Technology); phosphor-SAPK/JNK (Thr183/Tyr185) (1:1,000, #4668, Cell Signaling Technology); P38 MAPK (1:1,000, #9212, Cell Signaling Technology); phospho-P38 MAPK alpha (1:1,000, #4511, Cell Signaling Technology); GSK-3β (1:1,000, #12456, Cell Signaling Technology); phospho-GSK-3β (Ser9) (1:1,000, #9323, Cell Signaling Technology); β-catenin (1:1,000, #8480, Cell Signaling Technology); phospho-β-catenin (Ser33/37/Thr41) (1:1,000, #9561, Cell Signaling Technology); and β-actin (1:2,000, ab8227, Abcam) followed by peroxidase-conjugated secondary antibodies including polyclonal goat anti-rabbit (1:2,000, P0448, Dako); rabbit anti-goat (1:2,000, P0160, Dako), and polyclonal goat anti-mouse (1:2,000, P0447, Dako) in 2.5% non-fat milk (T145.1, ROTH) for 1 h at room temperature. The band signal detection was then performed with X-ray Amersham Hyperfilm (28906836, GE Healthcare) utilizing Amersham ECL Western Blotting Detection Reagents (9838243, GE Healthcare) and detected with an OPTIMAX X-Ray Film Processor (11701-9806-3716, PROTEC GmbH) in a dark room.

### Immunofluorescence Staining

Cementoblasts were cultured on sterile Falcon^TM^ Chambered Cell Culture Slides (354108, Fisher Scientific) until 50% confluence and afterward were fixed with 4% paraformaldehyde (Cat: 158127, Sigma-Aldrich) dissolved in 1X phosphate-buffered saline (PBS) (1401683, Gibco), adjusted to pH 7.4, for 10 min at room temperature, and permeabilized with 0.5% Triton^TM^ X-100 Surfact-Amps^TM^ Detergent Solution (28313, Thermo-Fisher) for 20 min. Then, cells were kept in a blocking buffer containing 10% goat serum, 0.3 M glycine, 1% BSA (071M8410, Sigma-Aldrich), and 0.1% Tween-20 (P1319, Sigma-Aldrich) for 30 min at room temperature and incubated with primary antibodies for GSK-3β (1:500, #12456, Cell Signaling Technology) and β-catenin (1:500, #8480, Cell Signaling Technology) at 4°C overnight. After washing three times with 1X PBS (1401683, Gibco)–0.1% TRITON X-100 (T-9284, Sigma-Aldrich) for 5 min, the cells were incubated with DyLight 488 goat anti-rabbit polyclonal secondary antibody (1:1,000, ab96899, Abcam), which conjugated to fluorescein isothiocyanate for 1 h. After washing with 1X PBS-Tween-20, DNA was stained using a fluorescent mounting medium with 4′,6-diamidino-2-phenylindole (DAPI) (ab104139, Abcam) for 15 min. Staining was analyzed using a high-resolution fluorescence microscope (Leica Microsystems, Wetzlar, Germany) and photographed.

### Dual Luciferase (Firefly–Renilla) Assay

OCCM-30 cells were seed into a 96-well plate at a density of 3 × 10^4^ cells per well in 100 μl growth medium [α-MEM (11095-080, Gibco) containing 10% FBS (10270-106, Gibco) and 1% penicillin/streptomycin (15140-122, Gibco)] overnight. The reverse transfection of OCCM-30 cells with silencing RNA was performed with specific siRNAs to knock-down *AdipoR1*, *AdipoR2*, *MAPK1*, *MAPK3*, *MAPK8*, and *MAPK14*. Briefly, cells were incubated with 24.25 μl Opti-MEM antibiotic-free medium (31985-062, Gibco) and 0.75 μl HiPerFect^@^ Transfection Reagent (301705, QIAGEN) supplemented with 12.5 ng specific siRNA for incubation for 24 h. The cells were simultaneously transfected with the TCF/LEF Reporter Kit (#60500, BPS Bioscience). For control transfection, cells were transfected with DNA mixture by 1 μl of TCF/LEF luciferase reporter vector (#60500, BPS Bioscience) plus negative control siRNA (1027280, QIAGEN); 1 μl of non-inducible luciferase vector (#60500, BPS Bioscience) plus negative control siRNA (1027280, QIAGEN); and 1 μl of non-inducible luciferase vector (#60500, BPS Bioscience) plus specific siRNA in 15 μl Opti-MEM antibiotic-free medium (31985-062, Gibco). For experimental transfection, the DNA mixture of 1 μl TCF/LEF luciferase reporter vector (#60500, BPS Bioscience) plus specific siRNA was incubated in 15 μl Opti-MEM antibiotic-free medium (31985-062, Gibco). All the DNA mixtures were then mixed with 0.35 μl Lipofectamine^TM^ 2000 Transfection Reagent (11668030, Thermo Fisher) in 15 μl Opti-MEM antibiotic-free medium (31985-062, Gibco) at room temperature for 25 min. After 24 h of transfection, the medium was changed to fresh growth medium. Following incubation for another 23 h, adiponectin (100 ng/ml) was added to stimulate cells for 1 h. We set up each treatment in triplicate.

After 48 h of transfection, the firefly luciferase activities were performed using the BPS Dual Luciferase (Firefly/Renilla) Assay system (#60683-1, BPS Bioscience) using a Mithras LB 940 Luminometer (38099, Berthold Technologies) and analyzed by MikroWin 2000 (Mikrotek Laborsysteme GmbH), which normalized to the Renilla luciferase activities.

### Statistical Analysis

Statistical analyses were plotted using the GraphPad Prism 6.0 software (GraphPad Prism Inc., San Diego, CA). Quantitative values are expressed as means ± standard deviation (SD) and analyzed using independent one-way *t*-test followed by Tukey’s *post-hoc* test for unpaired samples to determine the statistically significant differences for multiple comparisons. Differences were considered statistically significant at a *p*-value of < 0.05 and *p*-values are shown with respect to controls, unless otherwise indicated. Data distribution was analyzed using the Kolmogorov–Smirnov and the Shapiro–Wilk test and visually using QQ plots. All experiments were successfully performed in triplicate.

## Results

### Adiponectin or Compressions Up-Regulate the Expression of Adiponectin Receptors, MAPKs, and β-Catenin

**FIGURE 1:**
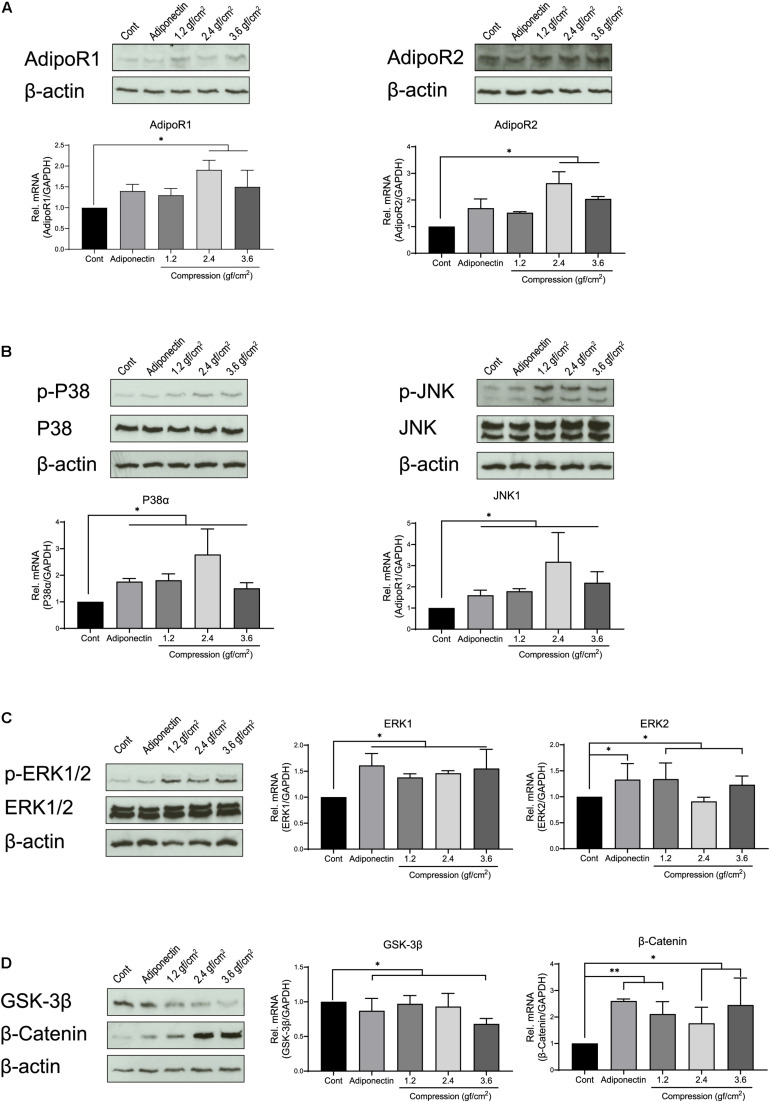
Adiponectin as well as compression regulates the expression of adiponectin receptors (AdipoRs), mitogen-activated protein kinase (MAPK), and β-catenin. **(A)** Representative western blots showing the expression changes of AdipoR1 and AdipoR2 in the presence of adiponectin (100 ng/ml) or compression (1.2, 2.4, and 3.6 gf/cm^2^). Real-time reverse transcriptase–polymerase chain reaction (RT-PCR) analysis shows that 2.4 or 3.6 gf/cm^2^ compressive forces significantly increase mRNA expression of adiponectin receptors (*AdipoR1* and *AdipoR2*) in mouse OCCM-30 cells (^∗∗^*p* < 0.01). **(B,C)** Western blots showing the expression of p-P38, p-ERK1/2, and p-JNK induced by adiponectin or compression. **(D)** The OCCM-30 cells show down-regulated mRNA expression of *GSK-3*β when exposed to adiponectin (100 ng/ml) or compression (3.6 gf/cm^2^), whereas up-regulated mRNA expression of β-*catenin* (^∗∗^*p* < 0.01). β-Catenin protein expression was increased after compression stimulation (^∗^*p* < 0.05). Graphics show mRNA expression levels of *P38*α, *JNK1*, *ERK1, ERK2, GSK-3*β, and β-*catenin* after 60-min stimulation with adiponectin or compression. Data were derived from three independent experiments. Data is normalized to 1, and values are expressed as means ± SD. Asterisks indicate significant differences compared to control cells (^∗∗∗^*p* < 0.001, ^∗∗^*p* < 0.01, and ^∗^*p* < 0.05; ns, not significant).

First, we investigated the expression of adiponectin receptors (AdipoRs) at mRNA and protein levels on OCCM-30 cells. To evaluate the effect that adiponectin as well as compressive forces exert *in vitro* on cementoblasts, cells were additionally stimulated with 100 ng/ml adiponectin. Next, we performed a dynamic analysis of receptor expression, cultivating the cells in the presence of different compressive forces: OCCM-30 cells underwent compressive forces of 1.2, 2.4, and 3.6 gf/cm^2^. The RT-PCR analysis revealed that the addition of adiponectin did not significantly influence *AdipoRs* mRNA expression (*p* > 0.05), while the application of compressive forces of 2.4 or 3.6 gf/cm^2^ significantly increased its expression (^∗^*p* < 0.05; Figure 1A).

Furthermore, we analyzed the relative mRNA expression of *P38*, *JNK*, and *ERK*. RT-PCR results show that the expression of *P38*α and *JNK1* was strongly up-regulated by adiponectin (^∗^*p* < 0.05) or compression (^∗^*p* < 0.05), whereas cells showed a slight up-regulation (^∗^*p* < 0.05) of *ERK1* and *ERK2* in the presence of adiponectin or compressive forces (Figure 1C). Adiponectin exerts a negative effect on the *GSK-3*β mRNA expression (^∗^*p* < 0.05), while it positively up-regulates β-*catenin* expression (^∗∗^*p* < 0.01) after 1 h of stimulation (Figure 1D). Compressive forces alone decrease the mRNA expression of *GSK-3*β (3.6 gf/cm^2^, ^∗^*p* < 0.05) and increase β-*catenin* expression (1.2 gf/cm^2^, ^∗∗^*p* < 0.01; 2.4 and 3.6 gf/cm^2^, ^∗^*p* < 0.05; Figure 1D).

Western blot assays show increased expression of p-P38, p-ERK1/2, and p-JNK as a reaction to adiponectin or compression (Figures 1A–D). These observations prompted us to examine the possibility that adiponectin or compression regulates MAP kinase and β-catenin on OCCM-30 cells.

### Adiponectin in Combination With Compression Promotes MAPK Signaling Activation

**FIGURE 2:**
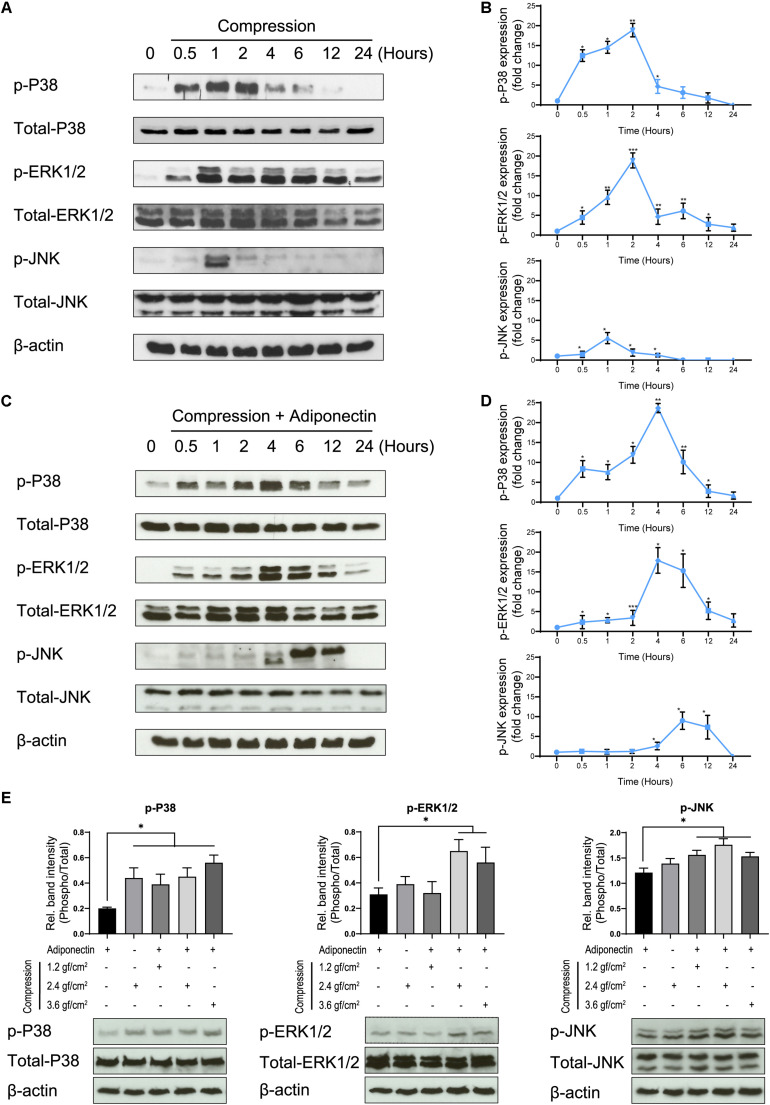
Adiponectin in combination with compression enhances MAPK signaling activation. **(A,B)** Compression promotes P38, ERK1/2, and JNK phosphorylation on OCCM-30 cells. The kinetic protein expression of P38, ERK1/2, and JNK as well as their phosphorylated forms in response to compressive forces of 2.4 gf/cm^2^ were analyzed by western blots. β-Actin served as a loading control. **(C,D)** After stimulation with 2.4 gf/cm^2^ compression and adiponectin (100 ng/ml), phosphorylated forms of P38, ERK1/2, and JNK were up-regulated at different time points. Graphics represent the relative expression values of p-P38, p-ERK1/2, and p-JNK normalized to control cells at time point 0 as protein fold changes, respectively. **(E)** Western blot showing the expression changes of MAPK protein induced by adiponectin (100 ng/ml), compression (2.4 gf/cm^2^), or adiponectin combined with compression (1.2, 2.4, and 3.6 gf/cm^2^). The quantification ratio of p-P38, p-ERK1/2, and p-JNK is shown as phosphorylated state unit/total unphosphorylated protein (Phospho/Total). The statistical analysis was based on three independent experiments. Values are shown as the means ± SD. Asterisks indicate significant differences compared to control cells (***p < 0.001, **p < 0.01, and *p < 0.05; ns, not significant).

Compressive forces of 2.4 gf/cm^2^ cause the phosphorylation of P38 and ERK1/2 after 30 min of cell exposure, whereas the phosphorylation of JNK occurs 1 h after stimulation (Figures 2A,B).

The co-cultivation of cementoblasts with adiponectin (100 ng/ml) under compressive forces (2.4 gf/cm^2^) resulted in an increased and sustained phosphorylation stage of P38, ERK1/2, and JNK, at different degrees. Western blots (WBs) revealed that the phosphorylation of P38 and ERK1/2 was maintained from 0.5 to 24 h, reaching a peaking during 2–6 h of stimulation. The phosphorylation of JNK occurred after 4 h, reaching a peak at time point 6 h (Figures 2C,D).

Furthermore, we found that adiponectin combined with compressive force enhances the protein level of p-P38, p-ERK1/2, and p-JNK on cementoblasts (Figure 2E).

### The Co-stimuli of Adiponectin With Compressive Forces Enhance β-Catenin Expression on Cementoblasts

**FIGURE 3:**
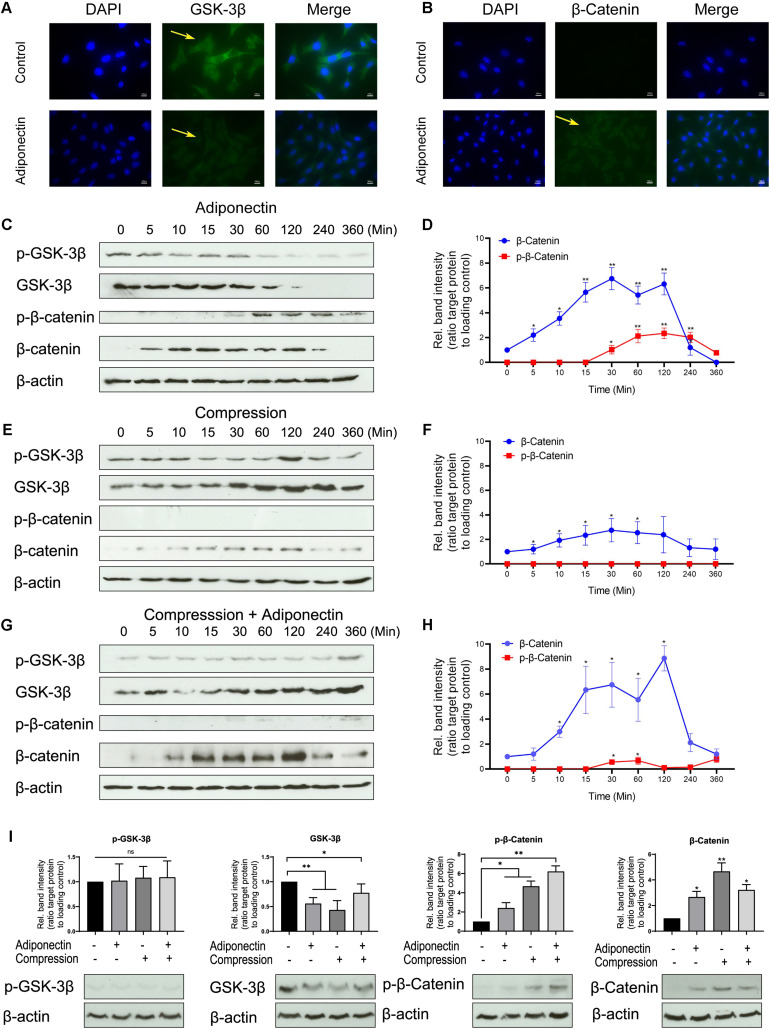
The co-stimuli of adiponectin with compression enhance β-catenin expression on cementoblasts. **(A,B)** Immunofluorescence staining shows that adiponectin (100 ng/ml) decreases the expression of cytoplasmic GSK-3β in OCCM-30 cells and the cellular β-catenin expression was increased after adiponectin (100 ng/ml) addition. **(C,D)** Western blots indicate that after adiponectin (100 ng/ml) addition to OCCM-30 cells, phosphorylated and total GSK-3β protein expression decreased after 1 h, whereas the expression of total β-catenin increased from 5 min to 2 h due to adipokine stimulation. **(E,F)** Compressive forces exert a dual effect on OCCM-30 cells, increasing the expression of GSK-3β as well as the levels of total β-catenin: western blots indicate that the expression of phosphorylated and total GSK-3β as well as total β-catenin is increased after exposure to 2.4 gf/cm^2^ compression for 0–6 h. **(G,H)** Compression in combination with adiponectin (100 ng/ml) enhances the expression of β-catenin: the kinetic analysis performed on OCCM-30 cells cultivated under 2.4 gf/cm^2^ compression combined with adiponectin (100 ng/ml) shows that total β-catenin was significantly up-regulated after 10 min of stimulation over a period of 4 h. **(I)** Representative western blot showing the expression changes of GSK-3β and β-catenin induced by adiponectin (100 ng/ml) in the presence or absence of compression (2.4 gf/cm^2^). Graphic represents the protein intensity, which was quantified as ratio to loading control to show the protein expression of p-GSK-3β, GSK-3β, p-β-catenin, and β-catenin. Data were derived from three independent experiments. Values are expressed as means ± SD. Asterisks indicate significant differences compared to control cells at time point 0 (***p < 0.001, **p < 0.01, and *p < 0.05; ns, not significant).

Immunocytochemistry staining revealed that adiponectin-mediated GSK-3β expression was almost fully inhibited after 30 min and β-catenin showed an increasing expression 30 min after stimulation (Figures 3A,B).

WB analysis shows an attenuated activity of GSK-3β phosphorylation and decreased GSK-3β expression in response to adiponectin (100 ng/ml) over a time period from 30 min to 3 h (Figures 3C,D). Adiponectin also enhances β-catenin phosphorylation after 1-h stimulation, but increases β-catenin accumulation concurrently from 5 min to 2 h (Figures 3C,D). Surprisingly indeed, we found that adiponectin administration caused a constant increase in the β-catenin expression, the time course of which was coincident with the acute and transient increase of β-catenin phosphorylation in OCCM-30 cells (Figures 3C,D).

Next, we performed WBs to verify the effect that compressive forces (2.4 gf/cm^2^) alone can have on the cellular accumulation of β-catenin. The time course experiments showed that the activation of β-catenin protein was transiently increased during 10 min to 2 h during compression exposure (Figures 3E,F), a fact that indicates that the application of compressive forces initially promotes WNT-independent accumulation of β-catenin in OCCM-30 cell cultures.

Additionally, we observed that adiponectin (100 ng/ml) in combination with compressive forces (2.4 gf/cm^2^) upregulates the expression of dephosphorylated β-catenin 10 min after exposure. This effect was sustained over a period of 3 h (Figures 3G,H). Furthermore, we found that OCCM-30 cultivated with adiponectin and compression showed a decreasing expression of GSK-3β and increasing expression of β-catenin (Figure 3I).

### Blockade of MAPKs Alters the Adiponectin and Compression-Induced Activation of β-Catenin on OCCM-30 Cells

**FIGURE 4:**
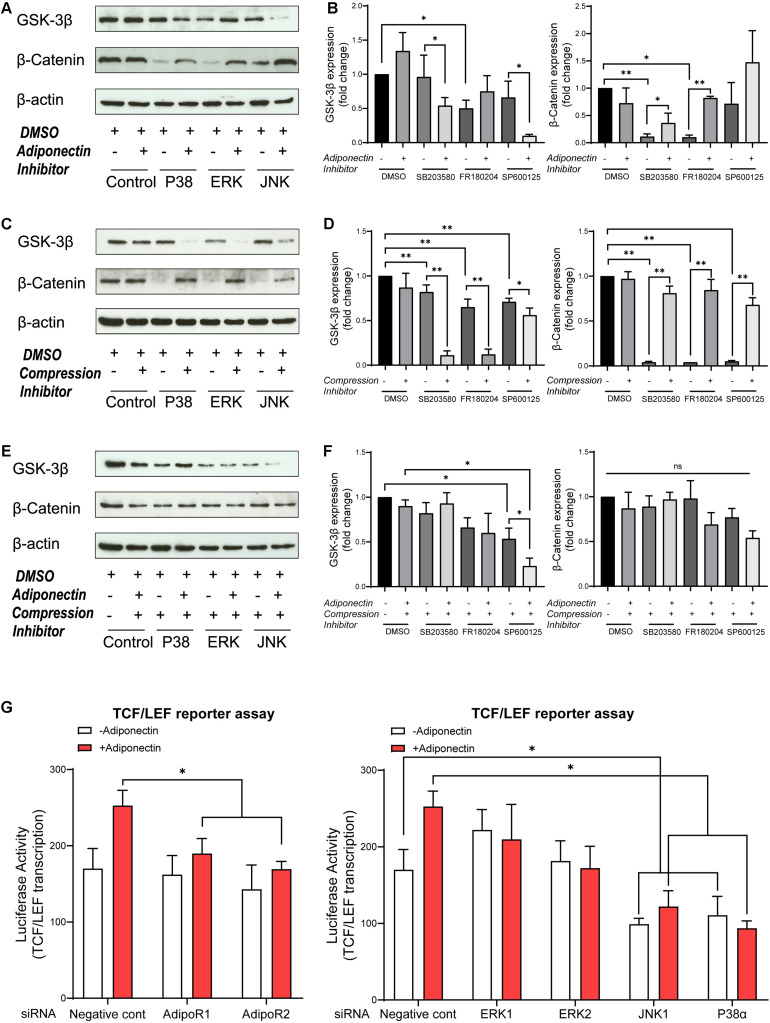
MAPK inhibition blocks β-catenin, whereas adiponectin addition effectively rescues its expression. **(A,B)** Western blots indicate that GSK-3β expression was influenced by MAPK inhibitors 1 h after adiponectin addition: pictures show that addition of SB203580 (P38) and SP600125 (JNK) inhibitors to OCCM-30 cells up-regulate GSK-3β protein expression. The expression of β-catenin was reduced after P38 and ERK1/2 inhibition. The suppressed β-catenin signaling could be rescued by adiponectin (100 ng/ml) in different degrees. **(C,D)** Compressive forces of 2.4 gf/cm^2^ decrease the expression of total GSK-3β protein in cells pretreated with SB203580 (P38), SP600125 (JNK), and FR180204 (ERK1/2) inhibitors and increase the expression of cellular β-catenin. Single suppression of P38, ERK, and JNK blocks β-catenin, but its expression was rescued by compression stimulation. **(E,F)** The negative effect that ERK1/2 and JNK inhibition exerts on the expression of total GSK-3β protein on OCCM-30 cells exposed to compression is not altered by adiponectin addition for 1 h of co-stimulation. P38 inhibitor combined with compression decreases the expression of GSK-3β. This effect was enhanced by adiponectin addition. The expression of β-catenin was not significantly altered in the presence of MAPK inhibitors and compression despite adiponectin. Graphics show the variations of GSK-3β and β-catenin protein expression as fold change when cells were exposed to MAPK inhibitors in cells cultivated under compressive forces (2.4 gf/cm^2^) and/or adiponectin (100 ng/ml) compared to controls. **(G)** OCCM-30 transfected with siRNA (*AdipoR1*, *AdipoR2*, *P38*α, *JNK1*, *ERK1*, and *ERK2*) as well as TCF/LEF luciferase reporter vector were subsequently treated with adiponectin for 1 h. Asterisks indicate statistical significance (^∗∗∗^*p* < 0.001, ^∗∗^*p* < 0.01, and ^∗^*p* < 0.05; ns, not significant).

Then, we examined whether adiponectin-mediated MAPK signaling activation may cross react with GSK-3β activity. In the absence of exogenous adiponectin, the blockade of ERK1/2 causes the total GSK-3β expression to decrease significantly, whereas by co-stimulation with adiponectin (100 ng/ml), such effect was reversed (Figure 4A). In the presence of adiponectin, the blockade of P38 and JNK decreases the total GSK-3β expression, indicating that adiponectin modulates GSK-3β via P38 and JNK signaling.

The expression of total β-catenin was down-regulated in the presence of P38 or ERK1/2 inhibitors, but rescued by adiponectin (100 ng/ml) addition, a fact that suggests that P38, ERK1/2, and JNK modulate GSK-3β and subsequent cellular events such as the cytoplasmic accumulation of β-catenin (Figures 4A,B).

In order to investigate whether the MAPK pathway influences β-catenin signaling during compression, the cells were cultivated under compressive forces of 2.4 gf/cm^2^ for 60 min. Afterward, we examined by WB the expression of GSK-3β and β-catenin. The results show that the suppression of the MAPK pathway has a significant blockade effect on β-catenin expression. This effect was restored when cells were exposed to compression for 1-h stimulation (Figures 4C,D). On the contrary, MAPK inhibition facilitates GSK-3β expression at varied degrees. These effects were counteracted by the application of compressive forces (Figures 4C,D).

Furthermore, to determine whether the activation of P38, ERK1/2, and JNK is involved in the stimulation of β-catenin by adiponectin or compression, the cells were preincubated with the pharmacological MAPK inhibitors SB203580 (P38), FR180204 (ERK1/2), and SP600125 (JNK) for 1 h and then cultivated under compressive forces of 2.4 gf/cm^2^ co-stimulated with adiponectin (100 ng/ml) for another 1 h.

As a result, we observed that ERK1/2 inhibition as well as JNK inhibition in combination with compression inhibit GSK-3β expression, whereas the total GSK-3β expression was promoted in the group treated with P38 inhibitor in the presence of compression and adiponectin (Figures 4E,F). The total expression of β-catenin was slightly reduced in all the groups treated with MAPK inhibitors and compression in the presence or absence of adiponectin in comparison to controls (Figures 4E,F), indicating that adiponectin modulates compression-induced GSK-3β and β-catenin expression partly throughout P38 MAPK signaling.

We next tested the effects of knocking down of *AdipoR1*, *AdipoR2*, *P38*α, *ERK1*, *ERK2*, and *JNK1* on adiponectin-stimulated TCF/LEF-sensitive transcription in cementoblasts (Figure 4G). Consistent with the effect on β-catenin accumulation, silencing *JNK1* and *P38*α attenuated the TCF/LEF reporter transcription (Figure 4G). The effect of silencing RNA targeting *AdipoR1* and *AdipoR2* results in a slight inhibition of TCF/LEF transcription, indicating the involvement of adiponectin receptors in this process (Figure 4G).

### Adiponectin Modulates GSK-3β and β-Catenin by AdipoR1 Commitment, and P38α and JNK1 Trigger β-Catenin Activation Due to Adiponectin Addition

**FIGURE 5:**
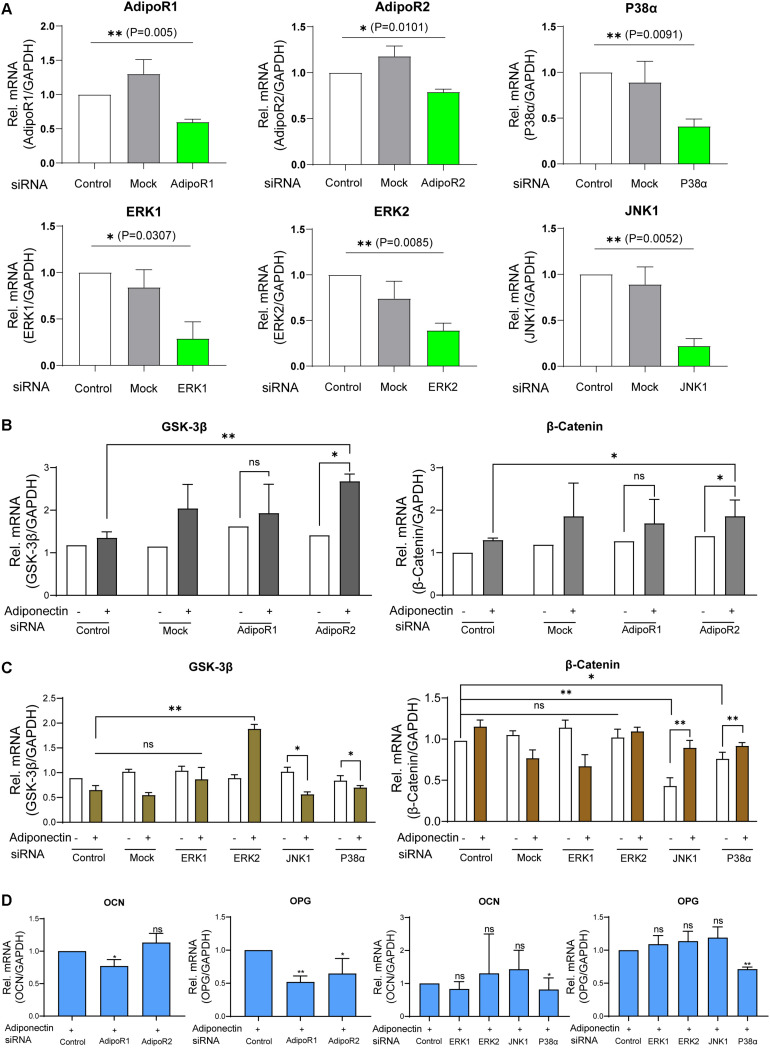
Adiponectin/AdipoR1/P38α cascade is particularly involved in adiponectin-induced cementogenesis. **(A)** The efficacy of siRNA transfections was analyzed by RT-PCR analysis. **(B)** Single knocking down of *AdipoR2* in the presence of adiponectin has a positive effect on *GSK-3*β gene expression and significantly increases β-*catenin* expression (^∗^*p* < 0.05). **(C)** The single silencing of *ERK2* causes increased gene expression of *GSK-3*β after adiponectin addition (^∗∗^*p* < 0.01). Silencing of *P38*α and *JNK1* slightly activates *GSK-3*β gene expression. After adiponectin addition, its expression was significantly decreased (^∗^*p* < 0.05). Single suppression of *P38*α or *JNK1* significantly decreased β-*catenin* expression (^∗∗^*p* < 0.01 and ^∗^*p* < 0.05, respectively), an effect that was restored after adiponectin addition in both groups. **(D)** Single knocking down of *AdipoR1* or *AdipoR2* has a down-regulating effect on *OCN* (^∗^*p* < 0.05) and *OPG* (^∗∗^*p* < 0.01) mRNA expression. The single silencing of P38α causes a significant down-regulation of *OCN* (^∗^*p* < 0.05) and *OPG* gene expression in the present of adiponectin (100 ng/ml) (^∗∗^*p* < 0.01). These effects were not observed by *ERK1*, *ERK2*, and *JNK1* single suppression. Asterisks indicate statistical significance (^∗∗∗^*p* < 0.001, ^∗∗^*p* < 0.01, and ^∗^*p* < 0.05; ns, not significant).

To define the individual contribution of the adiponectin receptors as well as the MAPK isoforms stimulated with adiponectin on β-catenin regulation, we silenced the mRNA expression of *AdipoR1*, *AdipoR2*, *ERK1* (*MAPK3*), *ERK2* (*MAPK1*), *JNK1* (*MAPK8*), and *P38*α (*MAPK14*) using siRNA transfection. The efficacy of the gene knock-down by siRNA transfections was analyzed by RT-PCR. After a transfection period of 48 h, we could observe effective down-regulation of the target genes (Figure 5A).

Next, we stimulated the cells with adiponectin (100 ng/ml), and we observed that the group treated with siRNA against *AdipoR2* showed significant increases of *GSK-3*β (^∗^*p* < 0.05) and β-*catenin* mRNA levels (^∗^*p* < 0.05), whereas mock treatment did not alter either *GSK-3*β or β-*catenin* mRNA levels significantly. Conversely, the group treated with siRNA against *AdipoR1* did not show significant differences in the expression of *GSK-3*β or β-*catenin* (Figure 5B).

After a 48-h incubation with siRNA against *ERK1*, *ERK2*, *JNK1*, and *P38*α mRNA or mock treatment in the presence or absence of adiponectin, RT-PCR was performed to analyze *GSK-3*β or β-*Catenin* mRNA expression. Adiponectin treatment in *JNK1* and *P38*α siRNA groups has a down-regulation effect on the *GSK-3*β mRNA expression. The silencing of *ERK2* but not that of *ERK1* causes the *GSK-3*β mRNA up-regulation after adiponectin addition (^∗∗^*p* < 0.01). The single knock-down of *JNK1* or *P38*α results in the reduction of β-*catenin* mRNA expression. This downregulation was rescued after adiponectin addition (100 ng/ml; Figure 5C).

After cementogenesis induction, knock-down of *AdipoR1* or *AdipoR2* decreased *osteocalcin* (*OCN*) and *osteoprotegerin* (*OPG*) mRNA expression at varying degrees (^∗^*p* < 0.05; Figure 5D). In the presence of adiponectin, the single silencing of *P38*α, but not that of *ERK1*, *ERK2*, or *JNK1*, significantly decreases *OCN* and *OPG* expression. The showed data indicate that the adiponectin/AdipoR1/P38α cascade is particularly involved in adiponectin-induced cementogenesis (^∗∗^*p* < 0.01).

## Discussion

In the present study, we demonstrated that adiponectin as well as compressive forces activate the MAPK and β-catenin pathways on cementoblasts. Through a cross-link mechanism, compression as well as adiponectin induce β-catenin accumulation on OCCM-30 cells by MAPK pathway commitment principally via AdipoR1. This effect involves multiple mediators including JNK1 and P38α that are key players in this process.

We observed that adiponectin can interact with cementoblasts and influence their biological response to compressive forces promoting the expression of p-P38, p-ERK1/2, and p-JNK. Interestingly, we found a longer up-regulation period of p-P38 and p-ERK1/2 by co-stimulation of adiponectin combined with compression, while the expression levels of p-JNK were altered after co-stimulation and reached an expression peak later. This indicates that adiponectin additionally triggers the MAP kinase pathway. In accordance with our previous data (2020), adiponectin strongly induced the expression of phosphorylated P38, ERK1/2, and JNK on cementoblasts (Yong et al., 2020).

The cooperative interaction between the β-catenin and MAPK signaling pathways has been reported in several studies (Bikkavilli et al., 2008; Thornton et al., 2008; Bikkavilli and Malbon, 2009; Osaki and Gama, 2013). On MC3T3-E1 cells, MEKK2 commitment promotes bone formation by rescuing β-catenin degradation (Greenblatt et al., 2016). Chen et al. (2010) reported that the blockade of P38 phosphorylation eliminates the activation of WNT signaling in preosteoblasts, indicating that osteoblast differentiation triggered by P38 MAPK/β-catenin promotes bone growth in female Sprague Dawley rats (Chen et al., 2010). Decreased phosphorylated P38 and increased ERK protein levels facilitate the β-catenin pathway through enhancing the expression of WNT3 and β-catenin in MC3T3-E1 cells (Guo et al., 2017). TGF-β-activated kinase 1 was reported to promote the phosphorylation of P38, JNK, and β-catenin, and at the same time, it down-regulated GSK-3β expression in mesenchymal stem cells (Yang et al., 2018). In contrast, Wang et al. (2012) showed that pretreatment with a P38 inhibitor (SB203580) on primary osteoblasts did not affect β-catenin protein expression (Wang et al., 2012).

In the present study, we observed that JNK, ERK1/2, as well as P38 chemical inhibition reduce β-catenin expression on OCCM-30 cells time dependently. However, the single suppression of JNK exerted a delayed effect on β-catenin reduction in comparison to cells treated with SB203580 (P38) and FR180204 (ERK1/2) antagonists. Furthermore, the single gene silencing of *ERK1* and *ERK2* as well as *P38*α and *JNK1* showed that adiponectin-induced β-*catenin* activation on OCCM-30 cementoblasts was especially sensitive to *P38*α and *JNK1* knock-down. *JNK1* and *P38*α silencing negatively regulates β-*catenin*, whereas *ERK1* has a significant positive effect on *GSK-3*β expression. Furthermore, β-*catenin* expression was up-regulated after adiponectin addition. In these contexts, our results reveal the role of both molecules triggering adiponectin–MAPK and β-catenin signaling interactions. Our data is consistent with previous studies (Thornton et al., 2008; Sakisaka et al., 2016). Thornton et al. (2008) showed that P38 MAPK inactivates GSK-3β, and this inactivation leads to an accumulation of β-catenin in the brain and thymocytes. Sakisaka et al. (2016) showed that P38 MAPK modulates β-catenin transcriptional activity, but has no effects on the phosphorylated GSK-3β as well as β-catenin expression in dental follicle cells. However, our present results do not clarify the relationship between the expression of GSK-3β and the phosphorylated status of MAPK signaling mediators (P38, JNK, and ERK1/2). Therefore, future studies should be performed to elucidate this mechanism in detail.

**SCHEME 1:**
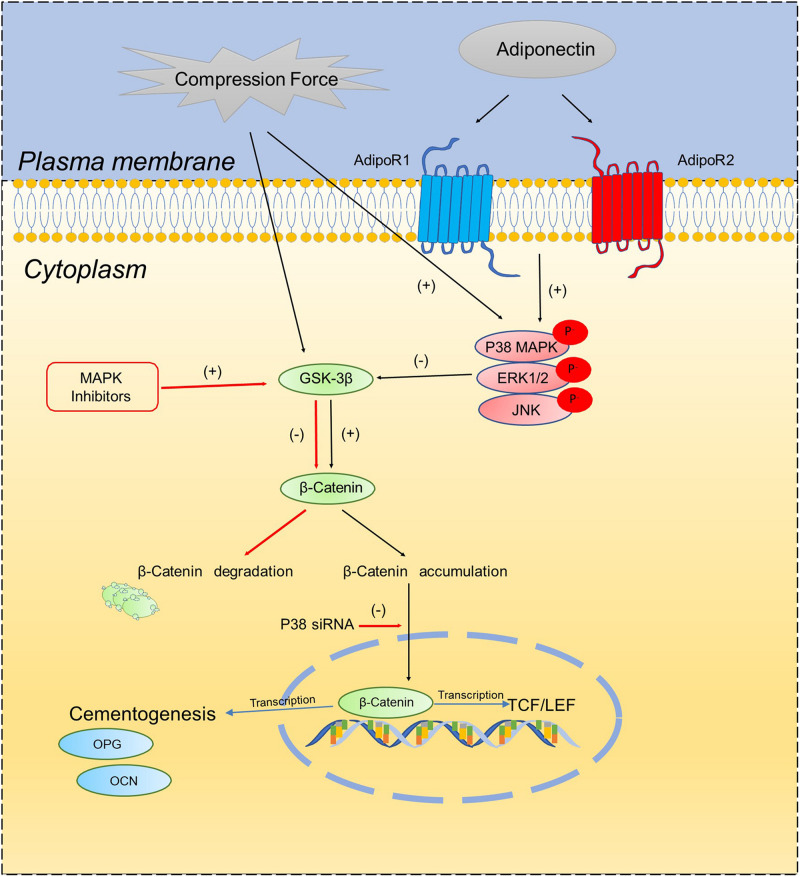
Schematic diagram illustrates the proposed molecular interactions between MAPK signaling pathways and β-catenin on OCCM-30 cementoblasts cultivated under compressive forces and adiponectin.

In the current study, we verified that compression forces activate β-catenin signaling in OCCM-30 cells. This finding is in accordance with the results of Shuqin et al. (2015) describing that the effect of mechanical strain on OCCM-30 cells regulate RUNX2 and β-catenin expression (Shuqin et al., 2015). Recently, Sindhavajiva et al. (2018) showed that compression induces human mandibular-derived osteoblast differentiation via WNT/β-catenin signaling. However, Korb et al. (2016) demonstrated the compression-induced apoptosis of human primary cementoblasts by up-regulation of the pro-apoptotic gene AXUD1 via a JNK-dependent pathway. Our study shows that the inhibition of β-catenin due to MAPK inhibition was restored by the application of light compressive forces on cementoblasts. Based on this observation, we conclude that MAPK activation is the bridge factor between compression application and β-catenin signaling activation. The proposed schema (Scheme 1) depicts that adiponectin activates β-catenin indirectly through the MAPK pathway. Compression forces induced an accumulation of β-catenin, whereas this process could be interacted by MAPK signaling especially through P38α.

An important observation that warrants discussion is that adiponectin addition combined with compression to OCCM-30 cells did not significantly affect β-catenin expression, even under the inhibition of MAPKs. This fact suggests that adiponectin addition exerts a multistep process in β-catenin modulation that needs to be further investigated. However, all present results imply that MAPK signaling participates in the process of the β-catenin signaling on cementoblasts.

Wang et al. (2017) reported *in vivo* and *in vitro* that adiponectin could facilitate bone mesenchymal stem cell (BMSC) osteogenic differentiation and osteogenesis by the WNT/β-catenin pathway. Nemoto et al. (2009) indicated that the inhibition of GSK-3β on cementoblasts suppresses alkaline phosphatase (ALP) activity and the gene expression of *ALP*, *bone sialoprotein* (*BSP*), and *osteocalcin* (*OCN*) but promotes cell proliferation.

In our experiment, knock-down of *AdipoR2* in OCCM-30 cells induces β-*catenin* up-regulation after adiponectin addition. Furthermore, our findings showing that the up-regulation of GSK-3β mRNA expression occurred after adiponectin addition in AdipoR2-silenced group strongly suggest that the modulation effect that adiponectin exerts on β-*catenin* expression is mostly mediated by AdipoR1. These data raise the possibility that adiponectin indirectly up-regulated β-catenin through the adiponectin/adipoR1 pathway.

*OCN* and *OPG* mRNA levels, which were identified as cementogenesis biomarkers (d’Apuzzo et al., 2013), were decreased after *P38*α knock-down in the presence of adiponectin. Hence, there is a possibility that *OCN* and *OPG* can be altered by the interplays between MAPK and β-catenin signaling in the cellular responses of cementoblasts under adiponectin and compression forces.

Clinical studies have described that the level of adiponectin is reduced in the serum of obese individuals (Hug et al., 2004). In this context, obese patients undergoing orthodontic treatment may be influenced by these lower levels of circulating adiponectin. As constant or intermittent mechanical compression is present on the stress side of the tooth root surface, we could speculate that less expression of adiponectin–MAPK signaling in this area leads to less activation of β-catenin. The possible clinical implication of this study may be that depressed adiponectin levels in obese subjects during orthodontic tooth movement may affect OIIRR in response to mechanical stimulation by decreasing β-catenin, which is capable of inhibiting mediators such as *OPG* and *OCN* that form extracellular matrices in OCCM-30 cells (Qiao et al., 2011). However, further biology response of the cross-talk network into how adiponectin effects such as promoting apoptosis should be identified.

## Conclusion

In conclusion, we demonstrated that adiponectin as well as compressive forces stimulate both the MAPK and β-catenin signaling pathways on cementoblasts. The inhibition of P38, JNK, and ERK1/2 differently regulates β-catenin expression as well as TCF/LEF transcription. Furthermore, adiponectin regulates the compression-induced β-catenin signaling via cross-interacting with the MAPK signaling pathway.

## Data Availability Statement

The raw data supporting the conclusions of this article will be made available by the authors, without undue reservation.

## Author Contributions

JY acquired and analyzed the data. JY, GR-H, and JB interpreted the data and wrote the manuscript. JY, GR-H, and SR conceived, designed, and supervised the study. All authors contributed to the article and approved the submitted version.

## Conflict of Interest

The authors declare that the research was conducted in the absence of any commercial or financial relationships that could be construed as a potential conflict of interest.
